# Social Technology in the Prevention of Adolescent Violence: documentary production

**DOI:** 10.1590/0034-7167-2023-0298

**Published:** 2024-11-22

**Authors:** Daiane de Paulo Paltanin Silva, Laura Christina Macedo, Rafaela Gessner Lourenço, Thammy Novakovski dos Santos

**Affiliations:** I Universidade Federal do Paraná. Curitiba, Paraná. Brazil

**Keywords:** Violence, Adolescent, Adolescent Health, Primary Health Care, Social Technology, Violencia, Adolescente, Atención Primaria de Salud, Salud del Adolescente, Tecnología Social

## Abstract

**Objective:**

To report the development of a technological innovation in the form of a documentary, aimed at disseminating actions for preventing violence against adolescents within the context of Primary Health Care.

**Methods:**

The documentary was developed through action research and interventions with adolescents in the territory of a Health Unit, conducted between 2020 and 2022, using Social Technology as both an action strategy and a conceptual reference.

**Results:**

The Social Technology proposal, created through workshops and seminars, resulted in a documentary focusing on violence prevention, involving 48 adolescents. The documentary’s script, collectively developed, portrays a soccer match between teams symbolizing peace and violence, with peace ultimately prevailing. The documentary is six minutes long.

**Final Considerations:**

The documentary has proven to be an effective Social Technology tool among adolescents, as it fosters critical thinking, is accessible, has potential for digital dissemination, and appeals to the target audience.

## RESUMEN

**Objetivo:**

Relatar el desarrollo de una innovación tecnológica en formato de documental para divulgar acciones de prevención de la violencia contra adolescentes en el escenario de la Atención Primaria de Salud.

**Métodos:**

Desarrollo de un documental mediante investigación-acción e intervenciones con adolescentes de un territorio de una Unidad de Salud, realizado entre 2020 y 2022, utilizando la Tecnología Social como estrategia de acción y marco conceptual.

**Resultados:**

La propuesta de Tecnología Social, construida en talleres de trabajo y seminarios, resultó en un documental sobre la prevención de la violencia, involucrando a 48 adolescentes. El guion del documental, desarrollado colectivamente, retrata un partido de fútbol entre los equipos de la paz y la violencia, donde prevalece la paz. El documental tiene una duración de seis minutos.

**Consideraciones Finales:**

El documental se reveló como una herramienta potente de Tecnología Social entre los adolescentes, ya que fomenta el pensamiento crítico, es accesible, tiene potencial de difusión en medios digitales y es atractivo para el público objetivo.

## INTRODUCTION

Approximately one billion children and adolescents worldwide are affected by some form of psychological, sexual, or physical violence each year. It is estimated that one in every two individuals between the ages of 2 and 17 years old experiences some type of violence, potentially leading to disabling injuries or exposure to the risk of death ^(1)^. Since the integration of violence-related issues into the Unified Health System (SUS) with the National Policy for Reducing Morbidity and Mortality from Accidents and Violence (PNRMAV) in 2001, the Health Assistance Network (RAS) has undergone restructuring to serve and rehabilitate victims of violence.

Primary Health Care (APS) is the principal access point to the RAS but faces challenges in reaching the adolescent population due to geographical and organizational barriers. It is crucial to strengthen public policies and reconsider health team practices to provide welcoming and quality care, emphasizing promotion and prevention measures for adolescents, aiming for improved life and health conditions in adulthood ^(2)^.

Healthcare professionals within the RAS must be aware of changes in health service delivery to align with the needs and requirements of communities, thereby offering increasingly qualified support. To effectively meet population needs and the policies for enhancing SUS services, it is essential for workers to expand their understanding of new products, technologies, and methodologies for care improvement, pursuing avenues that facilitate the provision of better and effective health practices ^(2)^.

In Colombo-PR, the location of this study, violence against adolescents is notably prevalent, underscoring the necessity for actions addressing the violence inflicted on this demographic. These initiatives must take into account the perceptions and viewpoints of adolescents regarding violence. From this perspective, Social Technology (TS) comprises products, techniques, and methodologies developed in collaboration with communities, providing practical solutions for significant social change ^(3)^.

Developing TS can be an effective practice in APS, aiming to tackle violence against adolescents by bringing them closer to health services, engaging them in recognizing daily occurrences of violence, and fostering a culture of non-violence. Guided by this objective, the central question of this research is: What is the most effective social technology to develop in APS for use with adolescents as a tool to promote violence prevention?

## OBJECTIVE

To report the development of a technological innovation in the form of a documentary, aimed at disseminating actions for preventing violence against adolescents within the context of Primary Health Care.

## METHODS

### Ethical Aspects

This study is a part of the master’s thesis titled “Social Technology and the Promotion of Violence Prevention Practices in Adolescence in Primary Care”^(4)^, undertaken within the Postgraduate Program in Health Care Practices at the Federal University of Paraná (UFPR). Conducted in line with Resolution No. 466 of 2012 from the National Health Council, it was approved by the UFPR Health Sciences Research Ethics Committee. Informed consent was obtained from participants over 18 years of age and from the parents or guardians of the adolescent participants. The adolescents signed an Assent Form. To ensure anonymity, the adolescents were identified using the names of soccer players they chose, and the social organization workers were referred to by the names of soccer coaches.

### Study Type

This report covers technological innovation concerning the development of an initiative to prevent violence against adolescents in the Primary Health Care (APS) setting. The innovation entailed developing a documentary through interventions with adolescents residing in an area served by a Health Unit, carried out from November 2020 to July 2022. The Consolidated Criteria for Reporting Qualitative Research (COREQ)^(5)^ guidelines were adhered to in both the design and development of the research.

### Methodological Procedures

The research, characterized by its participatory qualitative nature, adopted the concept of Social Technology (TS) as both an action strategy and a conceptual framework, viewed as a method developed through interaction with society, capable of being reapplied, and leading to social transformation^(3)^. The documentary was produced via discussion workshops and seminars implemented as part of the action research-a social approach grounded in empirical evidence, where researchers collaboratively and participatively address a collective problem ^(4)^.

For the action research development, the methodological framework proposed by Thiollent ^(6)^ was employed, comprising 12 interconnected steps: Exploratory Phase; Research Theme; Problem Statement; The Role of Theory; Hypotheses; Seminar; Field of Observation, Sampling, and Representativeness; Data Collection; Learning; Formal/Informal Knowledge; Action Plan; External Dissemination. All these phases were thoroughly completed during the research. In the seminars, 10 meetings took place involving those responsible for organizing, executing, and developing the social project, thereby guiding the creation of the documentary (Chart 1).

**Chart 1:** Seminars, Colombo, Paraná, Brazil, 2023

Timeline	Objective/Theme	Decisions Made
Seminar 1	Present the research proposal, epidemiological data, and raised hypotheses.	Participation in the research, support in data collection, and observation and supervision of working groups with adolescents.
Seminar 2	Discuss the challenges in obtaining a spontaneous/random sample of adolescents.	Choosing intentional sampling.
Seminar 3	Plan the working group with adolescents.	Conducting the workshop before sports activities, at the skate park next to the soccer field.
Seminar 4	First day of workshop.	Data collection.
Seminar 5	Second day of workshop.	Data collection.
Seminar 6	Debate data collected in the workshop.	Decisions regarding the documentary production.
Seminar 7	Debate data collected in the workshop.	Development of a semi-structured script.
Seminar 8	Construct the script.	Approval of the documentary script and scheduling of the filming.
Seminar 9	Capture images for the documentary.	Selection of the professional to be hired.
Seminar 10	Present a preview of the documentary.	Determination of the documentary’s theme music and approval of the content.

### Study Setting

The setting for the study was a third-sector organization that operates a voluntary social project offering soccer classes to children and adolescents in a community in Colombo-PR, Brazil.

### Data Source

Two groups of individuals participated in the research: five workers from the social organization and 11 adolescents involved in the soccer school activities of the social project. Participants were intentionally chosen based on their social representativeness in the project and their interest and availability to participate.

### Data Collection and Organization

Data collection involved using field observation diaries by the researchers, a semi-structured questionnaire for participant characterization, and a collective interview, known as a working workshop, where the theme was discussed, and the format for presenting the material for dissemination was defined.

In the semi-structured questionnaire developed by the researchers, it was possible to characterize the group of adolescents by sex, age, race, educational level, number of people and rooms in the residence, and the familial relationship among the residents. Additionally, a questionnaire with 25 questions, including seven from the CADRI scale (Conflict in Adolescent Dating Relationships Inventory) ^(7)^, was applied to identify the group’s perception of violence.

The workshops were audio-recorded and fully transcribed. From the discussions, it was decided that the material would be developed in a digital media format. For capturing images, following the semi-structured script, the soccer field where the training sessions took place was used. The team of volunteers selected the adolescents who would form the teams and the cheering section.

The adolescents prepared banners and rehearsed cheers for use during the match. The recording was conducted by a cinematographer experienced in institutional and advertising videos, using equipment such as a Sony A7III camera, lenses, DJI Ronin SC standard (China) image stabilizer, DJI Mini 2 SE version (China) drone, and lapel microphone. The images were edited in Adobe Premiere Pro version 22.2 (Brazil).

### Data Analysis

The data were analyzed using Bardin’s thematic content analysis methodology ^(8)^, which includes three phases: pre-analysis, material exploration, and treatment of results and interpretation. The pre-analysis involved the complete transcription of the speeches and an initial review of the document. In the second phase, exhaustive reading and coding were conducted based on the frequency and intensity of the speeches. Finally, semantic and lexical categorization was performed, considering the meanings and senses of the speeches, resulting in the emergence of the empirical category: “Social Technology in the form of a documentary as a tool for combating violence.”

## RESULTS

Regarding the workers from the social organization, the study included three men and two women, with ages ranging from 28 to 35 years. One professional had completed high school, two had some college education, and two had attained higher education, holding degrees in health and human sciences. Among the participating adolescents, there were six girls and five boys, aged between 10 and 15 years. In terms of ethnic self-identification, two identified as Black, five as Brown, and four as White. The educational levels of the adolescents ranged from the 5th grade of elementary school to the 1st year of high school. They reported living in homes with an average of four rooms, sharing the space with two other children. All resided with blood relatives and had diverse professional aspirations, including four who dreamt of a career in professional soccer.

In the adolescents’ statements, there was a clear condemnation of gender violence, in which the man is depicted as the aggressor and the woman as the victim. However, this disapproval seems to lessen when discussing physical violence among men:

*I myself heard some violence in the street by my house just this past night... Almost got up to... you know? You hear everything... during the night... The urge to help is strong, right? But then you never know what the situation is like.* (MBAPPÉ)

In the adolescents’ discourse, it is evident that they condemn gender violence, where the man is the aggressor and the woman is the victim. However, this disapproval disappears when they are questioned about physical violence among men, as highlighted in the following excerpt:

*So, you’re saying that a man hitting a woman is not right?* (RESEARCHER)

*Of course!* (MESSI)

*It’s never been right!* (MBAPPÉ)

*And a man hitting another man, is that right?* (RESEARCHER)

*Of course!* (MESSI)

*Ah, they can kill each other with punches, for all I care!* [makes a gesture of disdain with his hand and laughs]. (MBAPPÉ)

Despite recognizing situations of physical violence in their environment, the group was unable to present concrete solutions for such acts. However, they emphasized the need to invest in prevention and agreed that the best way to disseminate information would be through videos on online platforms, particularly social networks.

*A video would be better because most people don’t stop to read a text. I don’t stop to read text.* (MESSI)

*A YouTube channel is cool. Nowadays, most people have a cellphone.* [...] *Most people are just on social media. It’s better to post on social media, everyone is online.* (MBAPPÉ)

Therefore, the documentary script was collectively developed during the workshops. The adolescents decided to produce inclusive visual material, with translation into Brazilian Sign Language (LIBRAS), aiming to reach as many people as possible. This choice was encouraged by the presence of two adolescents who communicate using sign language in the researched social project. The content included the recording of short phrases or a poem created by the group.

[...] *short phrases, text, whatever. Could it be a poem, too? A poetry piece*. (RENARD)

Furthermore, it was agreed that the documentary’s story would depict a soccer match between the teams of peace and violence, with peace prevailing. The match would be narrated by an adolescent, who would present data on violence in the municipality before the start of the game.

[...] *we could organize a friendly match, then most of the kids would participate.* (ABEL FERREIRA)

*Good idea! And it could be about violence vs. peace.* (TITE)

*We could have one of them do a speech at the start of the video, like a presenter.* (GUARDIOLA)

Regarding the teams that played the match, participants chose to use words that denote violence or peace instead of player names. It was established that the players representing peace would be: friendship, dialogue, solidarity, support, school, protection network, family, affection, healthy fun, sports, and protection. Those representing violence would be: abandonment, bullying, aggression, drugs, child labor, insults, exclusion, self-harm, school dropout, hunger, unemployment, and teenage pregnancy. In terms of color representation, the team opted to use traffic light colors, deciding that positive words would be in green and negative words in red.

The documentary was filmed in a single morning and involved the participation of 48 adolescents. Those who participated in the scriptwriting workshops acted as players, totaling 13 people divided between the teams and judges. The other 35 adolescents, who did not participate in the workshops, acted as extras in the audience.

The script was adapted according to the progress of the filming, based on suggestions and input from the project organizers, the group of adolescents, and the professional responsible for the recording. The most significant change occurred during the selection of the players. A member of the violence team questioned whether they would represent “evil” until the end of the game. It was proposed that the players of the violence team would be expelled one by one and, upon being expelled, assume the role of another player from the team of peace, until all migrated to the team of peace.

After the conclusion of the recording, an evaluation seminar was held, which also resulted in the selection of the rap “Children’s Song,” addressing urban violence, performed by César MC and Cristal, as the documentary’s soundtrack. The final step was the decision on the title “IT WILL NEVER BE JUST SOCCER: ADOLESCENTS IN SEARCH OF PEACE.” The documentary, after the editing process and inclusion of credits, has a duration of six minutes and one second.

## DISCUSSION

In this study, the identification and normalization of violence in the daily lives of a group of adolescents were evident, as well as the adaptation of behaviors in pursuit of inclusion in social groups. The perception of violence as a natural phenomenon can be understood from the notion that neutrality is more comfortable than recognizing the injustice of what is happening ^(9)^.

In the adolescents’ discourse, there is clear disapproval of gender violence, where the man is the aggressor, and the woman is the victim. However, this disapproval diminishes when discussing physical violence among men. This reveals how the ideology of hegemonic masculinity, anchored in a model of masculinity associated with power, virility, and aggressiveness, significantly influences the construction of these young people’s identities. Thus, to overcome this scenario, the deconstruction of social gender is essential, a crucial step for transforming reality ^(9)^.

In this context, the third sector plays a vital role in forming partnerships with the health sector, creating an environment conducive to implementing actions aimed at social transformation, especially in preventing violence among adolescents. Networking and an intersectoral approach align with the National Health Promotion Policy guidelines, promoting personal, institutional, and social interactions that can positively impact the work of institutions and communities ^(10)^.

The development of the documentary thus highlighted the needs of the study’s participant group. Combining the creation of Social Technology (TS) with the adopted research method enabled the creation of permanent, current visual material with democratic and active participation from the adolescent population, considering that the primary goal of TS is to develop interactive methods with society that are replicable and result in social transformation ^(3)^.

Regarding the online dissemination of the documentary, it is understood that social media plays a significant role in identity construction, particularly among adolescents. These platforms act as a means of interaction among individuals with similar interests, influencing knowledge acquisition, emotional expression, and cultural formation. Moreover, young people constitute the majority of social media users, a trend that has intensified with the advent of mobile devices ^(11)^.

Social media are powerful tools for health education, offering speed and reach in information dissemination. Using Instagram® to disseminate scientific content bridges the virtual with the search for additional information, reducing the distance between the population and health services. For adolescents, social networks strengthen individual and collective empowerment, encourage critical thinking, and foster behavioral changes aimed at improving quality of life.

Videos and music, especially Hip Hop and rap, were the preferred formats for the young people in this study, touching their emotions and creativity and promoting reflections on social inequalities and societal transformation. The importance of Primary Health Care (APS) as a setting to identify vulnerabilities and promote health is emphasized, highlighting the role of health teams as facilitators of healthy practices in communities and promoters of everyday transformations ^(11)^. Therefore, it is essential that adolescents’ needs are recognized by health teams, and that they are encouraged to realize their potential. A study conducted in Recife - PE showed that adolescents wish to contribute to improving their living conditions but require institutional support to develop citizenship and confront social and health vulnerabilities ^(12)^.

In the context of violence, Social Technologies (TS) show positive results. A project in Bahia with men who perpetrated domestic violence led them to reflect on their actions and promote behavioral changes ^(9)^. Internationally, a notable TS developed by Canadian educators during the Covid-19 social isolation included community educational practices and building relationships with social organizations. These actions aimed to prevent domestic and gender violence, enhancing women’s safety in the territory, for instance, by creating code words for help requests in violence situations and ensuring a physical support point ^(13)^.

### Study Limitations

This study has several limitations. The primary one is the generalizability of the results to broader or different contexts, which is not achievable with the adopted method. Another limitation is the reliance on digital technologies and social media for disseminating the documentary, which limits its reach to segments of the population without regular access to the internet or social networks. Additionally, it is noteworthy that the final version of the documentary was not presented to the research participants, which could have prompted modifications and new discussions. Finally, although the intervention was innovative, it would benefit from longitudinal follow-up to assess and determine its real and sustained impact.

### Contributions to the Field of Nursing

This study is pioneering in the development of Social Technologies (TS) aimed at preventing violence among adolescents. By integrating social technology and digital media use in health education, the study expands the scope of nursing practice, underscoring the importance of innovative and interactive approaches in engaging youth. Furthermore, the TS in question can serve as a model for developing intersectoral actions between Primary Health Care (APS) and third-sector organizations targeting the adolescent audience.

## FINAL CONSIDERATIONS

Through the development of a documentary to disseminate actions for preventing violence against adolescents in the APS context, a TS was created that fosters critical thinking on the subject, is accessible, and can be disseminated via digital media, thus extending its reach to the adolescent public. The documentary, inspired by the language of adolescents and developed participatively, integrates the developed resource into a set of actions with the potential to connect the adolescent population with health services. It presents a promising approach to promote networking, particularly in partnership with third-sector institutions.

The involvement of the third sector in the community can aid the implementation of violence prevention actions, especially among adolescents, as social projects led by civil society organizations embedded in the communities themselves can inspire cultural changes and encourage social participation. The vulnerability of adolescents and the challenges in accessing health services underscore the need for alternative approaches in APS.

To encourage the coordination of the intersectoral network in pursuit of participatory practices for preventing violence in adolescence and promoting a culture of peace, the innovative documentary technology showcased in this study was displayed in the Municipal Health Councils, the Municipal Council for the Rights of Children and Adolescents, and the Pole of Evolution of Socio-Educational Measures in the municipality where the study was conducted.

## Data Availability

The documentary “It Will Never Be Just Soccer: Adolescents in Search of Peace” is registered with the National Cinema Agency (ANCINE) under the certificate of product no. B22-003555-00000 and is available for online access on the YouTube platform ^(14)^.
