# Supplementary material for: Social Technology in the Prevention of Adolescent Violence: documentary production
Source: Rev Bras Enferm. 2024 Nov 22;77(5):e20230298. doi: 10.1590/0034-7167-2023-0298 (PMC11653870; doi:10.1590/0034-7167-2023-0298)
Supplement: Translation

# Translation: Tecnologia Social na Prevenção da Violência na Adolescência: produção de um documentário

**DOI:** 10.1590/0034-7167-2023-0298pt

**Authors:** Daiane de Paulo Paltanin Silva, Laura Christina Macedo, Rafaela Gessner Lourenço, Thammy Novakovski dos Santos

**Affiliations:** I Universidade Federal do Paraná. Curitiba, Paraná. Brasil

**Keywords:** Violência, Adolescente, Saúde do Adolescente, Atenção Primária à, Saúde, Tecnologia Social

## Abstract

**Objetivo:**

Relatar o desenvolvimento de uma inovação tecnológica em formato de documentário para divulgar ações de prevenção à violência contra adolescentes no cenário da Atenção Primária à Saúde.

**Métodos:**

Desenvolvimento de um documentário por meio de pesquisa-ação e intervenções com adolescentes de um território de uma Unidade de Saúde, realizado entre 2020 e 2022, utilizando a Tecnologia Social como estratégia de ação e referencial conceitual.

**Resultados:**

A proposta de Tecnologia Social, construída em oficinas de trabalho e seminários, resultou em um documentário sobre a prevenção da violência, envolvendo 48 adolescentes. O roteiro do documentário, desenvolvido coletivamente, retrata um jogo de futebol entre os times da paz e da violência, onde a paz prevalece. O documentário tem a duração de seis minutos.

**Considerações Finais:**

O documentário revelou-se uma ferramenta potente de Tecnologia Social entre os adolescentes, pois incentiva o pensamento crítico, é acessível, tem potencial de divulgação nos meios digitais e é atrativo ao público-alvo.

## INTRODUÇÃO

Cerca de um bilhão de crianças e adolescentes são acometidos por algum tipo de violência psicológica, sexual ou física anualmente no mundo. Estima-se que uma em cada duas pessoas na faixa etária dos 2 aos 17 anos sofra algum tipo de violência, podendo levar a lesões incapacitantes ou estar exposta ao risco de morte ^(1)^. Desde a introdução da problemática da violência no Sistema Único de Saúde (SUS) com a Política Nacional de Redução da Morbimortalidade por Acidentes e Violência (PNRMAV) em 2001, a Rede de Assistência à Saúde (RAS) tem passado por reestruturações para atender e recuperar vítimas de violência.

A Atenção Primária à Saúde (APS) é a principal porta de entrada para a RAS, mas enfrenta desafios no acesso para a população adolescente, devido a barreiras geográficas e organizacionais. É fundamental fortalecer políticas públicas e repensar as práticas das equipes de saúde para oferecer uma atenção acolhedora e de qualidade, priorizando ações de promoção e prevenção para os adolescentes, visando melhores condições de vida e saúde na idade adulta ^(2)^.

Profissionais que compõem a RAS precisam se atentar às mudanças na prestação de serviços de saúde de modo a adequar às necessidades e exigências das comunidades, oferecendo uma assistência cada vez mais qualificada. Para responder de forma satisfatória às demandas e fragilidades da população, bem como às políticas de qualificação dos serviços prestados pelo SUS, é essencial que os trabalhadores ampliem o campo de visão para a compreensão de novos produtos, tecnologias e metodologias de qualificação do cuidado prestado, em busca de perspectivas que oportunizem a oferta de melhores e boas práticas em saúde ^(2)^.

No município de Colombo-PR, cenário deste estudo, a violência contra adolescentes apresenta elevada incidência, o que aponta a necessidade de proposição de ações sobre a situação da violência perpetrada nesta faixa etária da população. As ações precisam considerar as percepções e perspectivas do próprio público adolescente acerca da violência. Nessa perspectiva, a Tecnologia Social (TS) é uma categoria que engloba produtos, técnicas e metodologias criados em colaboração com comunidades e que oferecem soluções práticas para causar transformações sociais significativas ^(3)^.

A criação de uma TS pode se mostrar como uma boa prática na APS, uma vez que visa estimular o enfrentamento às violências contra adolescentes, por meio da aproximação dessa população com o serviço de saúde, seu engajamento no processo de reconhecimento das situações cotidianas violentas e fomento à cultura de não violência. Em busca de tal vinculação, a questão norteadora desta pesquisa foi: Qual a melhor tecnologia social a ser desenvolvida na APS, e utilizada junto aos adolescentes, como ferramenta para incentivo à prevenção de violência?

## OBJETIVO

Relatar o desenvolvimento de uma inovação tecnológica em formato de documentário para divulgar ações de prevenção à violência contra adolescentes no cenário da Atenção Primária à Saúde.

## MÉTODOS

### Aspectos Éticos

O estudo integra a dissertação de mestrado intitulada “Tecnologia Social e a Promoção de Práticas de Prevenção da Violência na Adolescência na Atenção Primária”^(4)^, desenvolvida no Programa de Pós-Graduação em Práticas do Cuidado em Saúde da Universidade Federal do Paraná (UFPR). Foi realizado de acordo com a Resolução nº 466 de 2012 do Conselho Nacional de Saúde e aprovado pelo Comitê de Ética em Pesquisa do Setor Ciências da Saúde da UFPR. Obteve-se o Consentimento Livre e Esclarecido dos participantes maiores de 18 anos e dos pais ou responsáveis pelos adolescentes participantes. Os adolescentes assinaram o Termo de Assentimento. Para manter o anonimato, os adolescentes foram identificados por nomes de jogadores de futebol escolhidos por eles, e os trabalhadores da organização social, por nomes de técnicos de futebol.

### Tipo de Estudo

Este é um relato de inovação tecnológica sobre o desenvolvimento de uma ação promotora de prevenção da violência contra adolescentes no cenário da APS. A inovação consistiu no desenvolvimento de um documentário, elaborado a partir de intervenções com adolescentes residentes em um território vinculado a uma Unidade de Saúde, realizadas entre novembro de 2020 e julho de 2022. Observaram-se os critérios estabelecidos para o *Consolidated Criteria for Reporting Qualitative Research* (COREQ) ^(5)^ na elaboração e no desenvolvimento da pesquisa.

### Procedimentos Metodológicos

A pesquisa, de caráter qualitativo participativo, adotou o conceito de Tecnologia Social (TS) como estratégia de ação e referencial conceitual, considerando-o um método desenvolvido em interação com a sociedade, passível de reaplicação e que resulta em transformação social ^(3)^. O documentário foi elaborado por meio de oficinas de discussão e seminários propostos na pesquisa-ação, uma abordagem social baseada em uma fundamentação empírica, onde os pesquisadores buscam a solução de um problema coletivo de maneira cooperativa e participativa ^(4)^.

Para o desenvolvimento da pesquisa-ação, seguiu-se o referencial metodológico de Thiollent^(6)^, com 12 etapas articuladas: Fase exploratória; Tema da Pesquisa; Colocação dos problemas; O lugar da teoria; Hipóteses; Seminário; Campo de observação, amostragem e representatividade; Coleta de dados; Aprendizagem; Saber formal/Saber informal; Plano de ação; Divulgação Externa. Todas essas etapas foram integralmente cumpridas durante a pesquisa. Nos seminários, realizaram-se 10 encontros com a participação de responsáveis pela organização, execução e desenvolvimento do projeto social, orientando a construção do documentário (Quadro 1).

**Quadro 1:** Seminários, Colombo, Paraná, Brasil, 2023

MOMENTO	OBJETIVO/ TEMA	DECISÕES TOMADAS
Seminário 1	Apresentar a proposta de pesquisa, os dados epidemiológicos e as hipóteses levantadas.	Participação na pesquisa, apoio na coleta de dados e na observação e supervisão de grupos de trabalho com adolescentes
Seminário 2	Discutir a dificuldade de adesão de adolescentes por amostra espontânea/aleatória.	Opção por amostragem intencional
Seminário 3	Planejar o grupo de trabalho com adolescentes.	Realizar a oficina antes das atividades esportivas, na pista de *skate* ao lado da quadra de futebol
Seminário 4	Primeiro dia de oficina	Coleta de dados
Seminário 5	Segundo dia de oficina	Coleta de dados
Seminário 6	Debater dados coletados na oficina	Decisões acerca da produção do documentário
Seminário 7	Debater dados coletados na oficina	Construção de roteiro semiestruturado
Seminário 8	Construir roteiro	Aprovação do roteiro do documentário e agendamento da gravação
Seminário 9	Captar as imagens para o documentário	Definição do profissional a ser contratado
Seminário 10	Apresentar a prévia do documentário	Definição da música tema do documentário e aprovação do conteúdo

### Cenário do estudo

O cenário do estudo foi uma organização do terceiro setor, que promove um projeto social voluntário com aulas de futebol para crianças e adolescentes de uma comunidade no município de Colombo-PR.

### Fonte de Dados

Participaram da pesquisa dois grupos de indivíduos: cinco trabalhadores da organização social e 11 adolescentes envolvidos nas atividades da escola de futebol do projeto social. Os participantes foram intencionalmente escolhidos devido à sua representatividade social no projeto e ao interesse e disponibilidade para participar.

### Coleta e Organização dos Dados

A coleta de dados envolveu o uso de diários de observação em campo das pesquisadoras, um questionário semiestruturado para caracterização dos participantes e a realização de uma entrevista coletiva, chamada oficina de trabalho, onde se discutiu o tema e definiu-se a forma de apresentação do material para divulgação.

No questionário semiestruturado, desenvolvido pelas pesquisadoras, foi possível caracterizar o grupo de adolescentes quanto a sexo, idade, raça, escolaridade, número de pessoas e de cômodos na residência, e a relação de parentalidade entre os moradores. Além disso, aplicou-se um questionário com 25 questões, incluindo sete da escala CADRI (*Conflict in Adolescent Dating Relationships Inventory*) ^(7)^, com o objetivo de identificar a percepção do grupo sobre violência.

As oficinas foram gravadas em áudio e transcritas na íntegra. A partir das discussões, decidiu-se que o material seria desenvolvido em formato de mídia digital. Para a captação das imagens, seguindo o roteiro semiestruturado, utilizou-se a quadra de futebol onde ocorrem os treinos. A equipe de voluntários selecionou os adolescentes que formariam os times e a torcida.

Os adolescentes prepararam cartazes e ensaiaram gritos de torcida para usar durante a partida. A gravação foi feita por um cinegrafista experiente em vídeos institucionais e de propaganda, com equipamentos como câmera Sony A7III, lentes, estabilizador de imagem DJI Ronin SC *standard* (China), drone DJI Mini 2 versão SE (China) microfone de lapela. As imagens foram editadas no *Adobe Premiere Pro versão* 22.2 (Brasil).

### Análise dos Dados

Os dados foram analisados conforme a metodologia de análise de conteúdo temática de Bardin^(8)^, que inclui três fases: pré-análise, exploração do material, e tratamento dos resultados e interpretação. A pré-análise envolveu a transcrição integral dos discursos e leitura flutuante do documento. Na segunda fase, realizou-se a leitura exaustiva e codificação baseada na frequência e intensidade dos discursos. Finalmente, procedeu-se à categorização semântica e léxica, levando em conta os significados e sentidos dos discursos, resultando na emergência da categoria empírica: “Tecnologia social do tipo documentário como ferramenta para o enfrentamento da violência”.

## RESULTADOS

Em relação aos trabalhadores da organização social, participaram do estudo três homens e duas mulheres, com idades entre 28 e 35 anos. Um profissional tinha o ensino médio completo, dois possuíam ensino superior incompleto e dois tinham ensino superior completo, com formações nas áreas de saúde e ciências humanas. Quanto aos adolescentes participantes, foram seis meninas e cinco meninos, com idades variando entre 10 e 15 anos. Em termos de autodeclaração étnica, dois se identificaram como pretos, cinco como pardos e quatro como brancos. O nível de escolaridade dos adolescentes variou do 5º ano do ensino fundamental ao 1º ano do ensino médio. Eles relataram viver em residências com, em média, quatro cômodos, compartilhando o espaço com duas crianças. Todos moravam com parentes consanguíneos e tinham aspirações profissionais diversas, incluindo quatro que sonhavam com uma carreira no futebol profissional.

Nos discursos dos adolescentes, identifica-se uma condenação clara à violência de gênero, na qual o homem é o agressor e a mulher a vítima. No entanto, essa desaprovação parece desvanecer ao abordarem a violência física entre homens:

*Eu mesmo hoje essa madrugada escutei uma violência lá na rua da minha casa... Quase levantei pra... entendeu? Você já escuta tudo... durante a madrugada... A vontade de ajudar é grande né, mas daí vai saber como que tá.* (MBAPPÉ)

É possível identificar nos discursos que os adolescentes condenam a violência de gênero, em que o homem é o agressor e a mulher a vítima. No entanto, essa desaprovação desaparece ao serem questionados a respeito da violência física praticada entre homens, conforme trecho abaixo:

*Então você tá querendo dizer que o homem bater em mulher não está certo?* (PESQUISADORA)

*Claro!* (MESSI)

*Nunca teve certo!* (MBAPPÉ)

*E homem bater em homem, tá certo?* (PESQUISADORA)

*Claro!* (MESSI)

*Ah, podem se matar no soco, por mim!* [faz sinal de desdém com a mão e ri]. (MBAPPÉ)

Apesar de reconhecerem as situações de violência física em seu meio, o grupo não conseguiu apresentar soluções concretas para tais atos. Contudo, enfatizaram a necessidade de investir em prevenção, e concordaram que a melhor forma de disseminar informações seria por meio de vídeos em plataformas online, especialmente redes sociais.

*Um vídeo era melhor, porque a maioria não para pra ler um texto. Eu não paro pra ler texto.* (MESSI)

*Canal no youtube é massa. Hoje em dia a maioria do pessoal tem celular.* [...] *A maioria das pessoas tão só na rede social. Melhor postar na rede social, todo mundo tá online.* (MBAPPÉ)

Dessa forma, o roteiro do documentário foi desenvolvido coletivamente durante as oficinas. Os adolescentes decidiram produzir um material visual inclusivo, com tradução para a Língua Brasileira de Sinais (LIBRAS), visando atingir o maior número possível de pessoas. Essa escolha foi incentivada pela presença de dois adolescentes que se comunicam por linguagem de sinais no projeto social pesquisado. O conteúdo incluiu gravação de frases curtas ou um poema criado pelo grupo.

[...] *frases pequenas, texto, tanto faz. Pode ser um poema, também? Uma poesia.* (RENARD)

Além disso, acordou-se que a história do documentário representaria um jogo de futebol entre os times da paz e da violência, com a paz vencendo. A partida seria narrada por uma adolescente, que apresentaria dados sobre a violência no município antes do início do jogo.

[...] *podíamos fazer um amistoso, aí a molecada maioria participa.* (ABEL FERREIRA)

*Boa! E podia fazer da violência x paz.* (TITE)

*Poderia colocar um deles pra fazer uma fala no começo do vídeo, tipo um apresentador.* (GUARDIOLA)

No que se refere aos times que jogaram a partida, os participantes optaram pelo uso de palavras que denotam violência ou paz, em vez dos nomes dos jogadores. Foi estabelecido que os jogadores que representam a paz seriam: amizade, diálogo, solidariedade, apoio, escola, rede de proteção, família, carinho, diversão saudável, esporte e proteção. Já os que representam a violência seriam: abandono, bullying, agressão, drogas, trabalho infantil, xingamentos, exclusão, autoagressão, evasão escolar, fome, desemprego e gravidez na adolescência. Quanto à representação das cores, a equipe optou pelo uso das cores do semáforo, definindo que as palavras positivas estariam em verde e as negativas, em vermelho.

O documentário foi gravado em uma única manhã e contou com a participação de 48 adolescentes. Aqueles que participaram das oficinas de criação do roteiro atuaram como jogadores, totalizando 13 pessoas divididas entre os times e os juízes. Os outros 35 adolescentes, que não estiveram nas oficinas, atuaram como figurantes na torcida.

O roteiro foi adaptado de acordo com o andamento da gravação, com base nas sugestões e atitudes dos organizadores do projeto, do grupo de adolescentes e do profissional responsável pela gravação. A mudança mais significativa ocorreu durante a seleção dos jogadores. Um integrante do time da violência questionou se eles representariam “o mal” até o final do jogo. Surgiu a proposta de que os jogadores do time da violência fossem expulsos um a um e, ao serem expulsos, assumissem o papel de outro jogador do time da paz, até que todos migrassem para o time da paz.

Após a conclusão da gravação, realizou-se um seminário de avaliação, que também resultou na escolha do rap “Canção Infantil”, que aborda a violência urbana, interpretado por César MC e Cristal, como trilha sonora do documentário. A última etapa foi a decisão do título “NUNCA SERÁ SÓ FUTEBOL: ADOLESCENTES EM BUSCA DA PAZ”. O documentário, após o processo de edição e inclusão dos créditos, tem seis minutos e um segundo de duração.

## DISCUSSÃO

Neste estudo, foram evidenciados a identificação e a naturalização da violência no cotidiano do grupo de adolescentes, assim como a adaptação de comportamentos em busca de inclusão em grupos sociais. A percepção da violência como fenômeno natural pode ser compreendida a partir da ideia de que a neutralidade é mais confortável do que reconhecer como injusto o que está acontecendo^(9)^.

Nos discursos dos adolescentes, nota-se uma clara reprovação à violência de gênero, onde o homem é o agressor e a mulher, a vítima. Contudo, essa reprovação se atenua ao discutirem sobre a violência física entre homens. Isso revela como a ideologia da masculinidade hegemônica, ancorada em um modelo de masculinidade associado a poder, virilidade e agressividade, influencia significativamente na construção da identidade desses jovens. Assim, para superar esse cenário, é essencial a desconstrução social de gênero, crucial para a transformação da realidade ^(9)^.

Nesse contexto, o terceiro setor desempenha um papel vital na articulação e nas parcerias com o setor de saúde, criando um ambiente favorável para a implementação de ações voltadas à transformação social, especialmente na prevenção da violência entre adolescentes. A atuação em rede e a abordagem intersetorial estão em consonância com as diretrizes da Política Nacional de Promoção da Saúde, promovendo interações pessoais, institucionais e sociais que podem impactar positivamente no trabalho das instituições e das comunidades ^(10)^.

Portanto, o desenvolvimento do documentário deu visibilidade às necessidades do grupo participante do estudo. A associação da construção de uma Tecnologia Social (TS) com o método de pesquisa adotado possibilitou a criação de um material visual permanente, atual e com participação democrática e ativa da população adolescente, considerando que o objetivo principal da TS é desenvolver métodos interativos com a sociedade, que sejam replicáveis e resultem em transformação social ^(3)^.

Quanto à divulgação online do documentário, compreende-se que as mídias sociais desempenham um papel significativo na construção de identidades, especialmente entre os adolescentes. Estas mídias atuam como plataformas de interação entre indivíduos com interesses similares, influenciando a aquisição de conhecimento, a expressão de sentimentos e a formação cultural. Além disso, os jovens constituem a maioria dos usuários de redes sociais, uma tendência que se intensificou com o advento dos dispositivos móveis ^(11)^.

As mídias sociais representam ferramentas potentes para a educação em saúde, oferecendo rapidez e amplitude na disseminação de informações. O uso do Instagram® para divulgar conteúdo científico conecta o virtual à busca por informações adicionais, diminuindo a distância entre a população e os serviços de saúde. Para os adolescentes, as redes sociais reforçam o empoderamento individual e coletivo, incentivam o pensamento crítico e fomentam mudanças comportamentais que visam à melhoria da qualidade de vida.

Vídeos e músicas, especialmente Hip Hop e rap, foram os formatos preferidos pelos jovens no desenvolvimento do estudo, pois tocam suas emoções e criatividade, além de promover reflexões sobre desigualdades sociais e a transformação da sociedade. Destaca-se a importância da Atenção Primária à Saúde (APS) como cenário para identificar vulnerabilidades e promover saúde, ressaltando o papel das equipes de saúde como facilitadoras de práticas saudáveis nas comunidades e promotoras de transformações no cotidiano ^(11)^. Por isso, é essencial que as necessidades dos adolescentes sejam reconhecidas pelas equipes de saúde, e que eles sejam estimulados a perceber suas potencialidades. Um estudo realizado em Recife - PE mostrou que os adolescentes querem contribuir para a melhoria de suas condições de vida, mas precisam de suporte institucional para desenvolver cidadania e enfrentar vulnerabilidades sociais e de saúde ^(12)^.

No contexto da violência, as Tecnologias Sociais (TS) mostram resultados positivos. Um projeto na Bahia com homens que perpetraram violência doméstica os levou a refletir sobre seus atos e promover mudanças de comportamento ^(9)^. Internacionalmente, destaca-se uma TS desenvolvida por educadores canadenses durante o isolamento social na pandemia de COVID-19, que incluiu práticas educativas comunitárias e a construção de relações com organizações sociais. Essas ações visavam prevenir a violência doméstica e de gênero, aumentando a segurança das mulheres no território, por exemplo, através da criação de palavras-código para pedidos de socorro em situações de violência e da garantia de um ponto de apoio presencial ^(13)^.

### Limitações do Estudo

Este estudo apresenta limitações. A principal delas refere-se à generalização dos resultados para contextos mais amplos ou diferentes, o que não é possível utilizando o método adotado. Outra limitação é a dependência de tecnologias digitais e mídias sociais para a disseminação do documentário, restringindo o alcance a segmentos da população sem acesso regular à internet ou redes sociais. Além disso, destaca-se que o documentário finalizado não foi apresentado aos participantes da pesquisa, o que poderia ter gerado modificações e novas discussões. Por fim, a intervenção, embora inovadora, poderia se beneficiar de um acompanhamento longitudinal para avaliar e definir seu impacto real e sustentado.

### Contribuições para a Área da Enfermagem

Este estudo é pioneiro no desenvolvimento de Tecnologias Sociais (TS) voltadas à prevenção da violência entre adolescentes. Ao integrar a tecnologia social e o uso de mídias digitais na educação em saúde, o estudo expande o escopo de atuação dos enfermeiros, ressaltando a importância de abordagens inovadoras e interativas no engajamento juvenil. Ademais, a TS em questão pode servir como modelo para o desenvolvimento de ações intersetoriais entre a Atenção Primária à Saúde (APS) e organizações do terceiro setor voltadas ao público adolescente.

## CONSIDERAÇÕES FINAIS

Por meio do desenvolvimento de um documentário para divulgar ações de prevenção de violência contra adolescentes no cenário da APS, foi possível elaborar uma TS capaz de incentivar o pensamento crítico sobre o tema, acessível e com possibilidade de divulgação nos meios digitais, ampliando assim seu alcance entre o público adolescente. O documentário, inspirado na linguagem dos adolescentes e construído de forma participativa, insere o recurso desenvolvido num conjunto de ações com potencial de aproximar a população adolescente e os serviços de saúde. Trata-se de uma abordagem promissora para promover o trabalho em rede, sobretudo o desenvolvido em parceria com instituições do terceiro setor.

A participação do terceiro setor na comunidade pode favorecer a realização de ações de prevenção à violência, especialmente entre os adolescentes, pois projetos sociais liderados por organizações da sociedade civil, enraizadas nas próprias comunidades, podem ser inspiradores para promover mudanças culturais e incentivar a participação social. A vulnerabilidade dos adolescentes e as dificuldades de acesso aos serviços de saúde confirmam a necessidade de abordagens alternativas na APS.

Com o objetivo de fomentar a articulação da rede intersetorial em busca de práticas participativas de prevenção da violência na adolescência e para promover uma cultura de paz, a tecnologia inovadora do tipo documentário retratada neste estudo foi exibida nos Conselhos Municipais de Saúde, no Conselho Municipal dos Direitos da Criança e do Adolescente e no Polo de Evolução de Medidas Socioeducativas do município em que o estudo foi realizado.

## Data Availability

O documentário “Nunca será só futebol: adolescentes em busca da paz” está registrado na Agência Nacional do Cinema (ANCINE) sob o certificado de produto nº B22-003555-00000 e disponível para acesso online na plataforma Youtube^(14)^.
